# Mifepristone as a Potential Therapy to Reduce Angiogenesis and P-Glycoprotein Associated With Glioblastoma Resistance to Temozolomide

**DOI:** 10.3389/fonc.2020.581814

**Published:** 2020-10-05

**Authors:** Monserrat Llaguno-Munive, Sebastián León-Zetina, Inés Vazquez-Lopez, María del Pilar Ramos-Godinez, Luis A. Medina, Patricia Garcia-Lopez

**Affiliations:** ^1^Laboratorio de Farmacología, Subdirección de Investigación Básica, Instituto Nacional de Cancerología, Mexico City, Mexico; ^2^Posgrado en Ciencias Biomédicas, Universidad Nacional Autónoma de México, Mexico City, Mexico; ^3^Departamento de Patología Quirúrgica, Instituto Nacional de Cancerología, Mexico City, Mexico; ^4^Unidad de Investigación Biomédica en Cáncer INCan-UNAM, Instituto Nacional de Cancerología, Mexico City, Mexico; ^5^Instituto de Física, Universidad Nacional Autónoma de México, Coyoacán, Mexico City, Mexico

**Keywords:** glioblastoma, temozolomide, mifepristone, drug resistance, angiogenesis, P-gp

## Abstract

Glioblastoma, the most common primary central nervous system tumor, is characterized by extensive vascular neoformation and an area of necrosis generated by rapid proliferation. The standard treatment for this type of tumor is surgery followed by chemotherapy based on temozolomide and radiotherapy, resulting in poor patient survival. Glioblastoma is known for strong resistance to treatment, frequent recurrence and rapid progression. The aim of this study was to evaluate whether mifepristone, an antihormonal agent, can enhance the effect of temozolomide on C6 glioma cells orthotopically implanted in Wistar rats. The levels of the vascular endothelial growth factor (VEGF), and P-glycoprotein (P-gp) were examined, the former a promoter of angiogenesis that facilitates proliferation, and the latter an efflux pump transporter linked to drug resistance. After a 3-week treatment, the mifepristone/temozolomide regimen had decreased the level of VEGF and P-gp and significantly reduced tumor proliferation (detected by PET/CT images based on 18F-fluorothymidine uptake). Additionally, mifepristone proved to increase the intracerebral concentration of temozolomide. The lower level of O6-methylguanine-DNA-methyltransferase (MGMT) (related to DNA repair in tumors) previously reported for this combined treatment was herein confirmed. After the mifepristone/temozolomide treatment ended, however, the values of VEGF, P-gp, and MGMT increased and reached control levels by 14 weeks post-treatment. There was also tumor recurrence, as occurred when administering temozolomide alone. On the other hand, temozolomide led to 100% mortality within 26 days after beginning the drug treatment, while mifepristone/temozolomide enabled 70% survival 60–70 days and 30% survived over 100 days, suggesting that mifepristone could possibly act as a chemo-sensitizing agent for temozolomide.

## Introduction

Glioblastoma is the most frequent primary neoplasm of the central nervous system and the most aggressive brain tumor, with a life expectancy of 14–15 months post-diagnosis (1–3). It is characterized by uncontrolled cell proliferation, highly diffuse infiltration, resistance to apoptosis, robust angiogenesis, and DNA repair mechanisms contributing to drug resistance. The standard treatment for glioblastoma is surgery followed by chemotherapy based on temozolomide and radiotherapy, which leads to poor patient survival.

The growth of glioblastoma is associated with its capacity to maintain a balanced expression of proteins that control the cell cycle and allow for proliferation, motility and vascular neoformation. Furthermore, it is able to avoid recognition by the immune system. Reports in the Cancer Genome Atlas (TCGA) identify three main pathways participating in the pathogenesis of glioma: (RTK)/RAS/(PI3K), p53, and retinoblastoma (4).

A major factor in the strong resistance of tumors to temozolomide treatment is the overexpression of enzyme O6-methylguanine-DNA-methyltransferase (MGMT), which participates in the repair of temozolomide-induced DNA damage. Our group previously demonstrated that mifepristone enhances the temozolomide-induced decrease in orthotopic glioblastoma tumors by increasing apoptosis and reducing levels of MGMT (thus impeding repair of DNA damage) (5).

Among other pathways of glioma resistance to treatment described in the literature are those that contribute to angiogenesis, the formation of new blood vessels from a pre-existing vascular network. Several studies have correlated increased tumor vascularization with a lower rate of patient survival. Indeed, in the absence of angiogenesis, tumors cannot grow beyond a size of 1–2 mm^3^ (6). One of the main promoters of angiogenesis is hypoxia, which stimulates the synthesis of the most important mediator in angiogenesis, the vascular endothelial growth factor (VEGF). The receptors of VEGF are reported to be over-expressed in glioblastoma (7, 8). Among the strategies for inhibiting the expression of VEGF is the use of bevacizumab, a humanized monoclonal antibody. Two phase III studies on this drug have showed that the addition of bevacizumab to standard treatment (radiotherapy–temozolomide) for patients with newly diagnosed glioblastoma, was associated with a 4-month increase in progression-free survival without a significant effect on overall survival. Moreover, there was an increase in adverse events associated with bevacizumada (9, 10), emphasizing the need to seek new pharmacological strategies.

Another pathway involved in glioblastoma is related to the blood–brain barrier (BBB). Many promising chemotherapeutic agents have had great difficulty in overcoming the mechanisms of the BBB. On one hand, it is a physical barrier comprised of tight junctions between endothelial cells and a lack of fenestrae. In addition, it is an active efflux system that transports a wide range of antineoplastic drugs (e.g., temozolomide) out of the brain. The best known of these transporters is P-glycoprotein (P-gp), a membrane protein belonging to the superfamily of ATP-binding cassette (ABC) transporters. The blocking of these transport proteins might be useful in the treatment of glioblastoma (11–14).

To date, the search for new treatments against glioblastoma has not improved the survival of patients. An attractive strategy is the repositioning of approved drugs for use in combination with standard therapy. One attractive candidate for repositioning is mifepristone, a synthetic steroid that serves as an abortifacient drug based on anti-progestational and anti-glucocorticoid action. Mifepristone reportedly has antiproliferative effects in breast (15, 16), cervix (17), endometrium (18), ovary (19), and prostate cancer (20), can cross the BBB, and provides palliative effects on brain tumors such as meningiomas (21) and glioblastoma (22). Additionally, it is considered safe (with few adverse effects) and has a low cost. Besides reducing levels of MGMT (5), mifepristone is reported to diminish the activity of P-gp in human leukemia cancer cells (23) and a gastric cancer cell line (24). However, whether or not mifepristone is an inhibitor of P-gp on glioma cells or in the efflux transport system mediated by P-gp in the BBB has not yet been established. Likewise, there are no reports, to our knowledge, on its effect on temozolomide treatment.

Mifepristone may serve as a chemo-sensitizing drug, considering the descriptions in the literature of its inhibition of multiple targets in cancer cells. The aim of the present study was to evaluate the capacity of a mifepristone/temozolomide treatment in an orthotopic rat model of glioblastoma to modulate angiogenesis, reduce P-gp levels in the glioma tumors and increase the intracerebral concentration of temozolomide. Since tumors initially sensitive to chemotherapy often develop resistance, tumor recurrence was monitored after the combined treatment ended. Finally, the MGMT level was quantified as a parameter of DNA repair in tumor cells.

## Materials and Methods

### Drugs and Reagents

Mifepristone and temozolomide were provided by Sigma Chemical Co. (St. Louis, MO, United States). Dulbecco’s modified Eagle’s medium (DMEM), FBS (fetal bovine serum), and EDTA (Ethylenediaminetetracetic acid) were purchased from Gibco-BRL (Grand Island, NY, United States). LC-MS/MS grade methanol was acquired from J.T.Baker. Acetic acid was of analytical grade. High-quality water for the solutions was processed with a Milli-Q Reagent Water System (Continental Water Systems, El Paso, TX, United States). A stock solution of temozolomide was prepared in DMSO at a final concentration of 4% and mifepristone was reconstituted in polyethylene glycol/saline solution. All standard solutions were stored at −20°C until use.

### Animals

Male Wistar rats (230–250 g) were obtained from the Faculty of Medicine of the UNAM, Mexico City, Mexico. The animals were kept in pathogen-free conditions on a 12–12 h light/dark cycle, with adequate temperature and humidity. All procedures for the care and handling of the animals were reviewed and approved by the Ethics Committee of the “Instituto Nacional de Cancerología” (INCan, Mexico City, Mexico), (Ref. No. 010/17/IBI-CEI/601/10), and were in accordance with the Mexican Federal Regulation for Animal Experimentation and Care (NOM-062-ZOO-1999, Ministry of Agriculture, Mexico).

### Tumor Cell Implantation

The rat glioma C6 cell line was supplied by the American Type Culture Collection (ATCC, Rockville, United States). These cells were maintained under sterile conditions in DMEM medium (Gibco, Grand Island, NY, United States) supplemented with 5% fetal bovine serum and incubated at 37°C in a 5% CO_2_ atmosphere.

The effect of Mif/Tz on tumor growth was evaluated on C6 glioma cells orthotopically implanted in Wistar rats. Each animal was anesthetized with a combination of tiletamine hydrochloride (10 mg/kg) and acepromazine maleate (0.4 mg/kg) administered subcutaneously (sc), then placed in a stereotactic device for surgery. The tumor cell implantation was performed according to Llaguno et al. (5). Briefly, after fastening the head in the frame, a midline incision was made and bregma was identified. The skull was then drilled at the coordinates of 2.0 mm right from bregma and 6 mm deep (hippocampus). C6 cells were harvested, washed and diluted in DMEM to a concentration of 7.5 × 10^5^ in a volume of 3 μL. Employing an infusion pump, these cells were slowly implanted at a depth of 6 mm from the dura mater. The sham group was surgically opened and instead of implanting cancer cells, culture medium was injected.

### Treatments

At 2 weeks post-surgery, the rats were randomly divided into six groups: (A) negative control (without surgery and without treatment, (B) sham surgery (in the absence of glioma cells and drug treatments) and four groups with the surgical implantation of cancer cells: (C) without drug treatment (vehicle control), (D) temozolomide alone (Tz), (E) mifepristone alone (Mif), (F) mifepristone/temozolomide (Mif/Tz). Tz was administered at a dose of 5 mg/kg ip and Mif at a dose of 10 mg/kg sc. The drugs were given for five consecutive days (Monday–Friday) during 3 weeks.

### Determination of Tumor Growth

Brain tumor proliferation was measured by capturing images with a microPET/CT scanner (Albira ARS, Oncovision, Spain) at 2, 5, 7, 9, and 14 weeks post-surgery. For this purpose, 300 μCi of 18F-fluorothymidine (18F-FLT) were administered into the caudal vein. Another method of tracking tumor growth was by monitoring animal weight. Rats were weighed three times/week throughout the experiment, recording the global survival of each group.

### Histological Analysis

The rats were euthanized and perfused with saline solution followed by 4% paraformaldehyde. Brains were removed and immersed in 4% paraformaldehyde for 2 weeks. The brain tissue was embedded in paraffin and sliced into sections (2 mm thick) on the coronal plane for the subsequent analysis with Eosin and Hematoxylin (H&E) and microvessel density immunohistochemical was evaluated with CD31 marker (#77699, Cell Signalling Technology).

### Molecular Analysis

At the end of the study, the rats were sacrificed and the tumor was removed. The brain tissue was homogenized with a lysis buffer containing protease inhibitors (Cat. 78440; Thermo Scientist, TM). The samples were centrifuged at 10,000 *g* at 4°C and the supernatant was recovered. The proteins were quantified with the BCA (bicinchoninic acid) assay and separated by electrophoresis on 4–20% gradient gel (Mini-Protean TGX 456-1094, Bio-Rad Laboratories, Inc, United States). Colored markers (Bio-Rad, CA, United States) were included to establish size. For each sample, 40 g of protein were used. Following the transfer of the proteins onto PVDF membranes (Amersham, United Kingdom), the latter were blocked for 2 h at room temperature with 5% non-fat dry milk. The antibodies employed were anti-MGMT (sc-166528, 1:1000, Santa Cruz Biotechnology, TX, United States), P-gp (12683, 1:500, Cell Signalling Technology) and β-actin (sc-69879, 1:1000; Santa Cruz Biotechnology, TX, United States). After washing, the membranes were incubated with IRDye^®^ 800 CW goat anti-mouse or IRDye^®^ 680RD goat anti-rabbit secondary antibodies (1:15000; LI-COR, Inc.) for 1 h. The membranes were scanned on an Odyssey Imaging System and their intensity of fluorescence was measured with Image Studio software. In each figure, representative blot images were selected from the same gel. For the evaluation of angiogenesis, the relative concentration of VEGF was assessed with an Elisa kit according to the manufacturer’s instructions (human VEGF, ENZ-KIT156-0001, Enzo Life Sciences, Inc).

### Determination of Temozolomide in Rat Brain Tissue

Male Wistar rats (200–230 g) were divided into groups for two drug treatments (*n* = 6 each): (1) Tz (30 mg/kg, ip) and (2) Mif/Tz (60 mg/kg, sc, and 30 mg/kg, ip, respectively). For the second group, mifepristone was administered 2 h before temozolomide. In both groups, rats were euthanized 45 min after Tz was given. The tissues were weighed and kept at −70°C to await use.

The concentration of temozolomide was ascertained by chromatography on an LC-MS system (Agilent Agilent Technologies, Infinity 1260) with an autosampler temperature of 4°C. The separation was carried out at 25°C on an Agilent Zorbax SB-C18 column (1.8 μm, 2.1 mm × 50 mm) utilising a linear elution with (A) water (containing 0.5% acetic acid and 10 mM ammonium acetate) and (B) methanol as the mobile phase (10/90). The flow rate was set at 0.3 ml/min with an injection volume of 5 μl.

Mass spectrometry was performed on an Agilent QQQ Detector (Agilent Technologies, Infinity 1260) in the positive ESI mode with nitrogen as the solvent. The capillary voltage was 3.0 kV and the dissolvation temperature 350°C. Quantification was achieved by using multiple reactions monitoring of the transitions of *m*/*z* 195.10–137.95 for temozolomide, and *m*/*z* 181.10–124.0 for theophylline as the internal standard.

Individual stock solutions of temozolomide (1 mg/ml) and theophylline (1 mg/ml) were prepared in separate volumetric flasks and dissolved in acid methanol (acetic acid 0.5% and methanol v/v, 20/80) for temozolomide and pure methanol for theophylline. Intermediate and final working solutions containing temozolomide were prepared in acid methanol and theophylline solutions were prepared in water. Calibration standards were prepared at following concentrations: 50, 100, 500, 1000, 2000, and 5000 ng/ml.

The internal standard solution (1000 ng/ml in water) was added to small slices of the brain (400 mg; 50 μl 1 M HCL and temozolomide working solutions for the calibration standards). The slices were individually homogenized before adding ethyl acetate and mixing for 5 min. The samples were centrifuged at 14000 rpm for 15 min at 4°C. The supernatant was transferred to an Eppendorf tube, ethyl acetate was added, and centrifugation was performed at 14,000 rpm. The supernatant was transferred to an Eppendorf tube and evaporated to dryness under a stream of nitrogen at 24°C. Afterward, 200 μl of acid methanol was added to the dry residue and injected into the chromatographic system.

### Statistical Analysis

Data are expressed as the mean ± SD. Statistical significance was determined with one-way analysis of variance (ANOVA) on SPSS Base 20.0 software (SPSS Inc, Chicago, IL, United States). When necessary, the comparison of means was Bonferroni adjusted. In all cases, significance was considered at *p* < 0.05.

## Results

### Animal Body Weight

**FIGURE 1:**
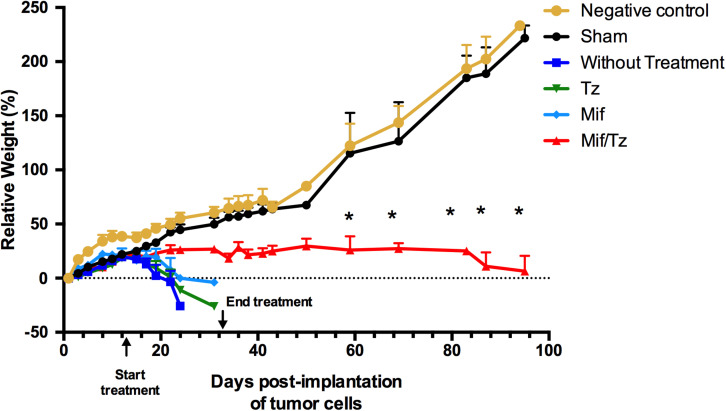
Tumor growth in the orthotopic rat model of glioma was evaluated by comparing animals weight between groups: negative control (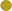) and sham surgery (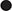); and in four groups with implanted glioma cancer cells, one without drug treatment (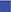) and the other given temozolomide only (Tz) (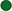), mifepristone only (Mif) (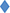), and mifepristone/temozolomide (Mif/Tz) (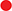). Each point of the graphic represents the mean ± SEM of six animals. *Significant difference (*p* < 0.05) between Mif/Tz and sham.

During the first 2 weeks post-implantation of C6 cells, all animals continued to gain weight. Subsequently, the negative control and sham group gained weight while the untreated, Tz and Mif groups rapidly lost weight, similar to data previously reported by our group (5). The rats in the Mif/Tz group maintained their weight throughout the experiment (Figure 1).

### Histological and Immunohistochemical Analysis

**FIGURE 2:**
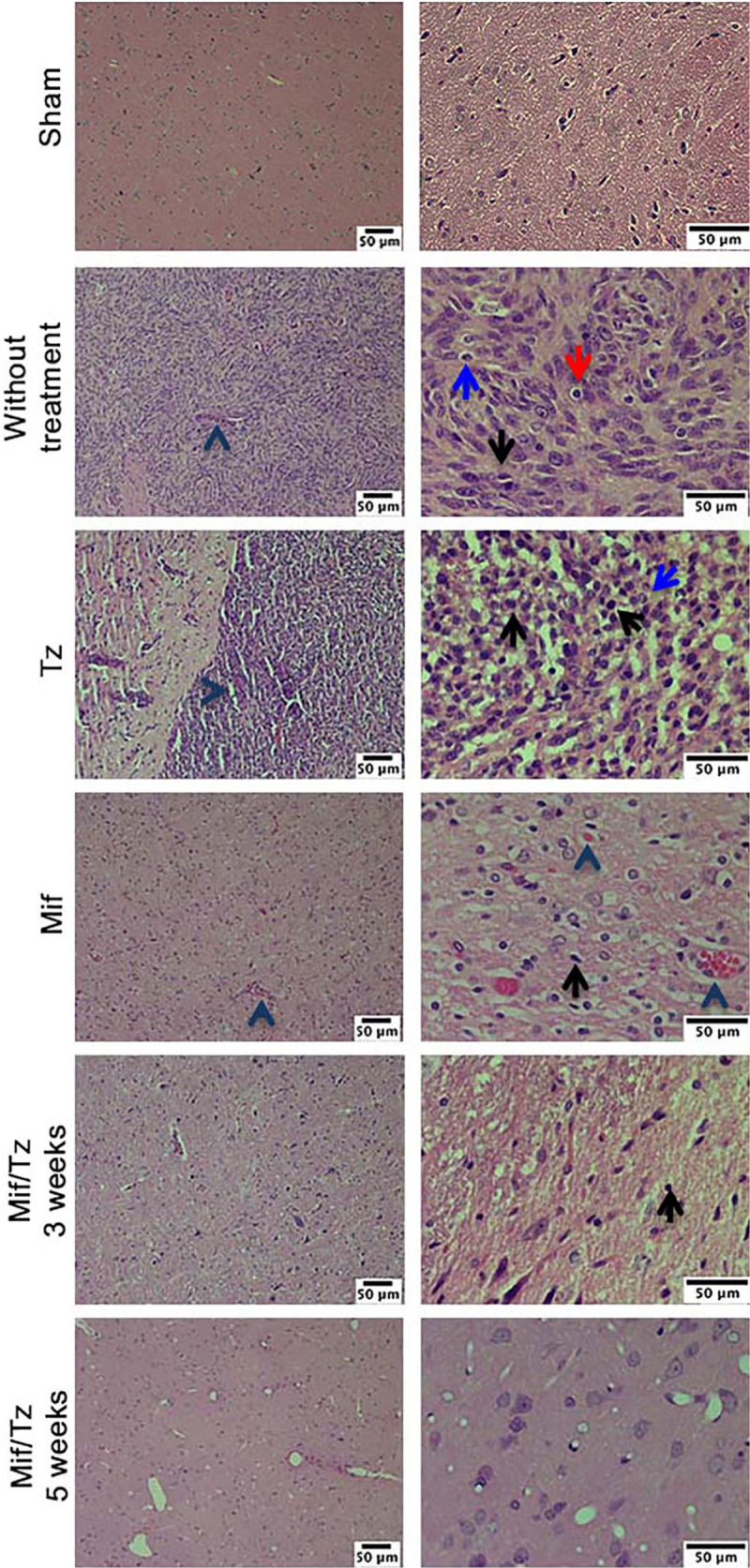
Hematoxylin and eosin (H&E) staining analysis of glioma tissue. Hyperbasophilic cells (black arrow), hyperchromatics cells (red arrow), vessel proliferation (arrowhead), mitosis (blue arrow). The images are representative of three animals per treatment Scale bars = 50 μm.

In the histological examination, applying H&E stain, we observed typical characteristics of glioblastoma in without treatment group, as hypercellularity, infiltration of tumor cells and mitosis. The tissue of the animals treated with Tz or Mif showed lesser hypercellularity and mitosis; however, the effect was more evident at 5 weeks post-surgery (at the end of the 3-weeks drug treatment period) with a considerable decrease infiltration of tumoral cells and inflammation cells, as well as the absence of pseudopalisading necrosis (Figure 2). These results are consistent with previously reported.

### Expression of VEGF

**FIGURE 3:**
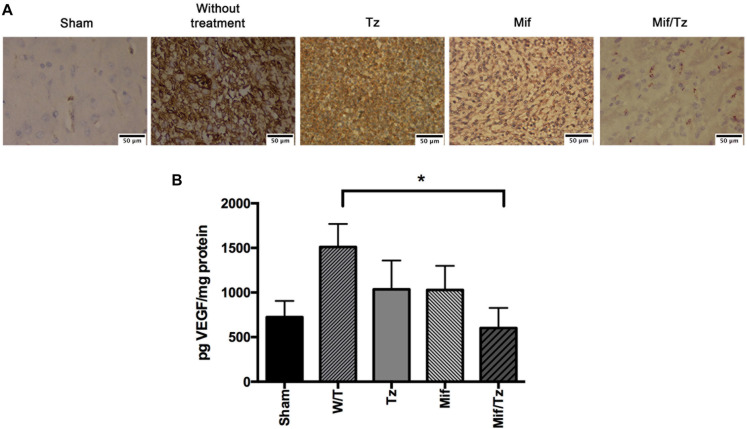
**(A)** Immunohistochemical staining of CD31 marker. Vessel density was assessed by immunostaining for CD31 positive glioma cell nuclei in rats implanted with glioma. The images are representative of three animals per treatment. Scale bar 50 μm. **(B)** Expression of VEGF at the end of the 3-week drug treatment, showing a significantly lower level in the mifepristone/temozolomide (Mif/Tz)-treated group versus the untreated (W/T) group, both with implanted cancer cells. Data are expressed as the mean ± SD from eight independent experiments. *Significant difference (*p* < 0.05) between the Mif/Tz and W/T group.

At the end of 3 weeks drug treatment the rats were sacrificed to evaluated CD31 marker and VEGF expression. Vascular density was determined by CD31 marker, we observed that Mif and Tz decrease the vascular density compared to without treatment group; however, this decrease was greater in Mif/Tz group, these results were corroborated with the quantification of VEGF (Figure 3A). VEGF expression is closely related to angiogenesis. Compared to the sham group, the untreated animals with implanted cancer cells displayed a significantly higher level of VEGF. Compared to the latter group, the level of VEGF declined (but not significantly) in animals receiving either Tz or Mif, and was significantly lower in the Mif/Tz group (Figure 3B).

### Expression of P-gp

**FIGURE 4:**
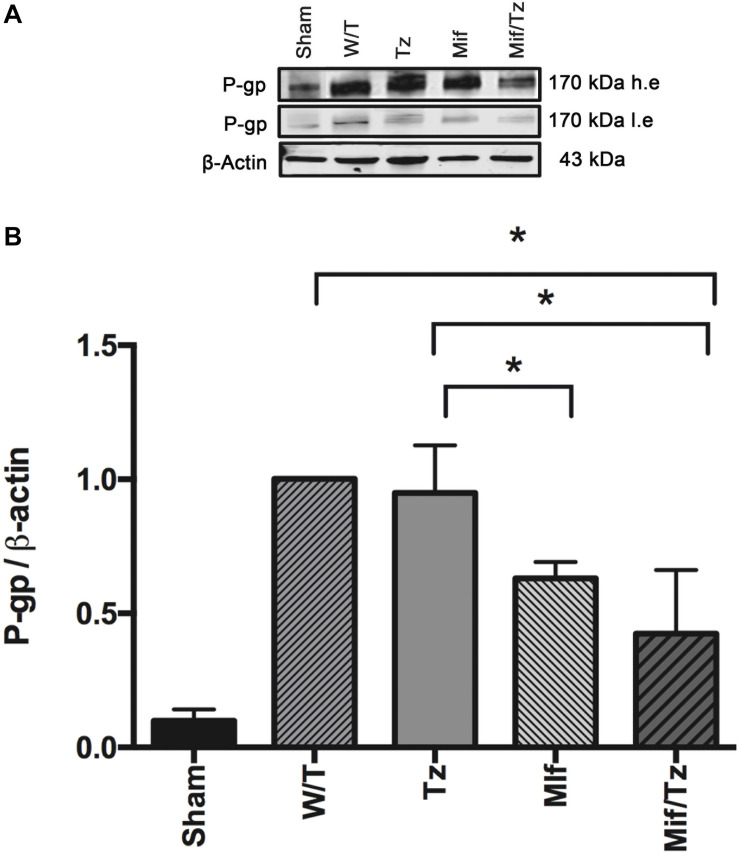
The quantification of P-gp levels at the end of the 3-week drug treatment evidenced a significant downregulation in the rats given mifepristone (Mif) or mifepristone/temozolomide (Mif/Tz) compared to those receiving no drug treatment (W/T) or temozolomide (Tz). **(A)** Representative Western blot; h.e., high exposure; l.e., low exposure. **(B)** densitometric analysis of the P-gp protein. Data are expressed as the mean ± SD from three independent experiments. *Significant difference (*p* < 0.05).

Western blot data and band intensity analysis revealed that the protein expression of P-gp (Figure 4) was downregulated at the end of the 3-week drug treatment (5 weeks post-surgery) in the Mif rats compared to the Tz and untreated groups. On the other hand, the Mif/Tz regimen caused an even greater reduction in this protein.

### Accumulation of Temozolomide in Brain Tissue

**FIGURE 5:**
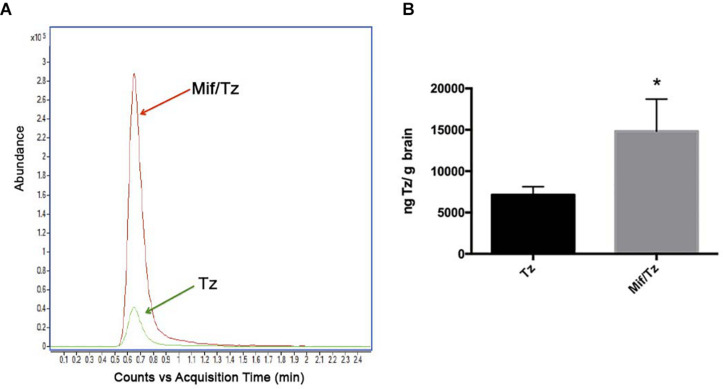
**(A)** Based on typical chromatograms of temozolomide in brain tissue, there was a significantly higher concentration of temozolomide (Tz) in rats given a pre-treatment of 60 mg/kg of mifepristone (Mif) followed by 30 mg/kg of Tz (red line) than in animals receiving only 30 mg/kg of Tz (green line). **(B)** Bar graph illustration of the Tz uptake in rat brain tissue (*n* = 6 ± SD). *Significant difference (*p* < 0.05).

The accumulation of temozolomide in brain tissue was determined by LC-MS analysis after treatment with Mif/Tz or Tz (Figure 5). Typical chromatograms obtained after the extraction of temozolomide in brain tissue from the groups of Tz and Mif/Tz are shown in Figure 5A. A significant two-fold greater intracerebral level of temozolomide was found in the brain tissue of the Mif/Tz versus Tz group (14820 ± 3852 vs 7136 ± 981 ng/g brain tissue); Figure 5B; *p* < 0.05.

### Therapeutic Effect of Mifepristone/Temozolomide on Tumor Size

**FIGURE 6:**
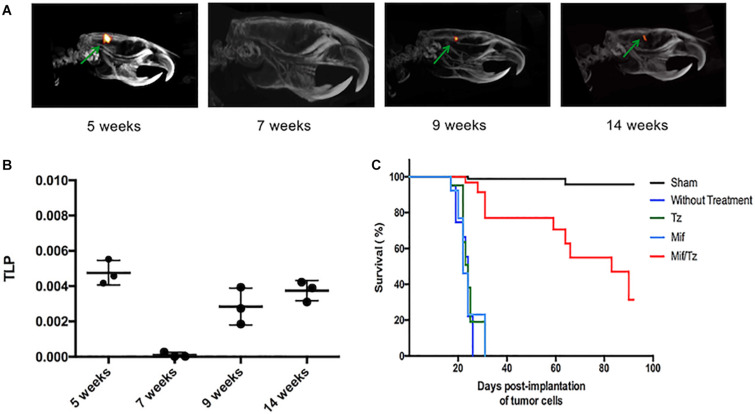
Proliferative activity in the orthotopic model of glioma evaluated by PET/CT images showing tumor uptake of 18F-FLT. **(A)** The images reveal the relative tumor size at 5, 7, 9, and 14 weeks post-surgery. Drug treatments were given from weeks 2–5. **(B)** The activity proliferative of tumors measured as total proliferation (TLP). **(C)** Survival analysis for 100 days after tumor cells implantation.

PET/CT scans were performed at 5, 7, 9, and 14 weeks post-implantation of tumor cells (the Mif/Tz treatments were given during week 2–5). In the images, the presence of red reflects the 18F-FLT uptake and thus the relative size of the tumor. The 18F-FLT uptake was higher at 5 weeks (3-week drug treatment). By 7 weeks post-surgery (2 weeks after the end of drug treatment), the 18F-FLT uptake had dropped drastically. At 9 weeks, however, 18F-FLT uptake appeared again, and can be observed at about the similar level at 14 weeks (Figure 6A). This suggests a tumor cell growth again at 9 weeks, indicating a possible tumor recurrence that remains stable at 14 weeks post-surgery. The 18F-FLT uptake was also measured as total lesion proliferation (TLP). At 7 weeks post-surgery a significant decrease of TLP was observed. Moreover, at 9 and 14 weeks post-surgery (4 and 9 weeks after the end of drug treatment), the TLP increased again (Figure 6B). The average survival time for rats was similar in the untreated, Tz or Mif groups, being 25–35 days. Contrarily, 70% of the Mif/Tz animals survived 60–70 days and approximately 30% survived over 100 days (Figure 6C).

### Histological Examination During Tumor Recurrence

**FIGURE 7:**
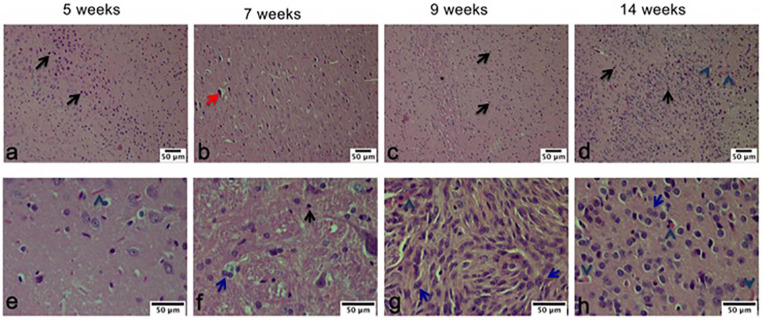
Hematoxylin and eosin (H&E) staining analysis of glioma tissue. The images are representative of three animals per treatment. Hyperbasophilic cells (black arrow), Hyperchromatics cells (red arrow), vessel proliferation (arrowhead), mitosis (blue arrow). Scale bars = 50 μm.

Within the pathological characteristics of glioblastoma are an increase of necrosis, mitosis, and pleomorphism as well as a vascularity proliferation. As shown in Figure 7, these characteristics decreased with the treatment of Mif/Tz (5 weeks), in the 7 weeks groups (2 weeks after the end of treatment) we observed some hyperchromatic cells and a decrease of hypercellularity; however, at 9 and 14 weeks pseudopalisading, necrosis, mitotic activity and vascular proliferation increased. A close correlation was observed with the molecular images of the same groups.

### Effect of Mifepristone/Temozolomide on VEGF During Tumor Recurrence

**FIGURE 8:**
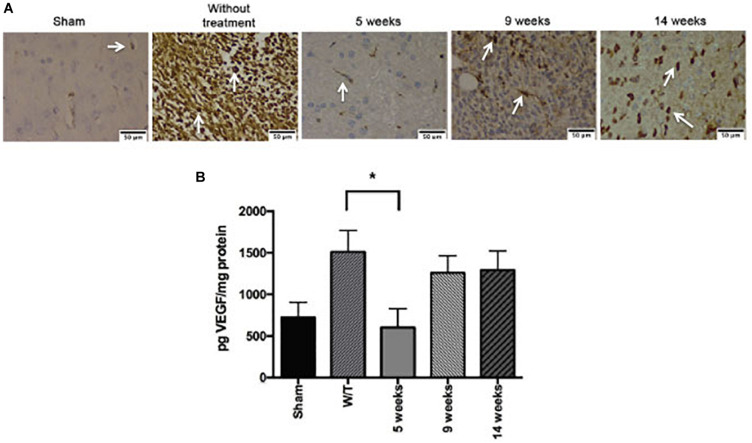
Effect of mifepristone/temozolomide on VEGF during tumor recurrence. **(A)** Immunohistochemical stainings with CD31 marker. Vessel density was assessed by immunostaining for CD31 positive glioma cell nuclei in rats implanted with glioma. The images are representative of three animals per treatment. Scale bar 50 μm. **(B)** Expression of VEGF in the sham-operated rats, implant-operated animals with no drug treatment (W/T), and at 5, 9 and 14 weeks post-surgery (the mifepristone/temozolomide (Mif/Tz) treatment were given only during weeks 2–5). Compared to the W/T rats, the Mif/Tz animals showed a lower level of VEGF at 5 weeks and a similar level at the time of tumour recurrence, at 9 and 14 weeks post-surgery. Data are expressed as the mean ± SD from five independent experiments. * Significant difference (*p* < 0.05) between the Mif/Tz and W/T group.

The brain tissue was processed for immunohistochemical assays with CD31 marker. At 5-weeks, Mif/Tz group showed a decrease in vessel density compared to without treatment group; however, there is an increase in positive cells at 9 and 14 weeks post-surgery (4 and 9 weeks after the end of drug treatment), interestingly, the density of positive cells was less compared to the group without treatment (Figure 8A). The VEGF levels at the end of the 3-week Mif/Tz treatment (at 5 weeks post-surgery) was significantly lower than that found in the untreated group and the same as that of the sham animals. However, this reduced level in the Mif/Tz group was reversed after drug treatment ended, during tumor recurrence at 9 and 14 weeks post-surgery (4 and 9 weeks after the end of drug treatment), this parameter increased in the Mif/Tz group, being similar to the value of the untreated group (Figure 8B).

### Effect of Mifepristone/Temozolomide on P-gp Levels During Tumor Recurrence

**FIGURE 9:**
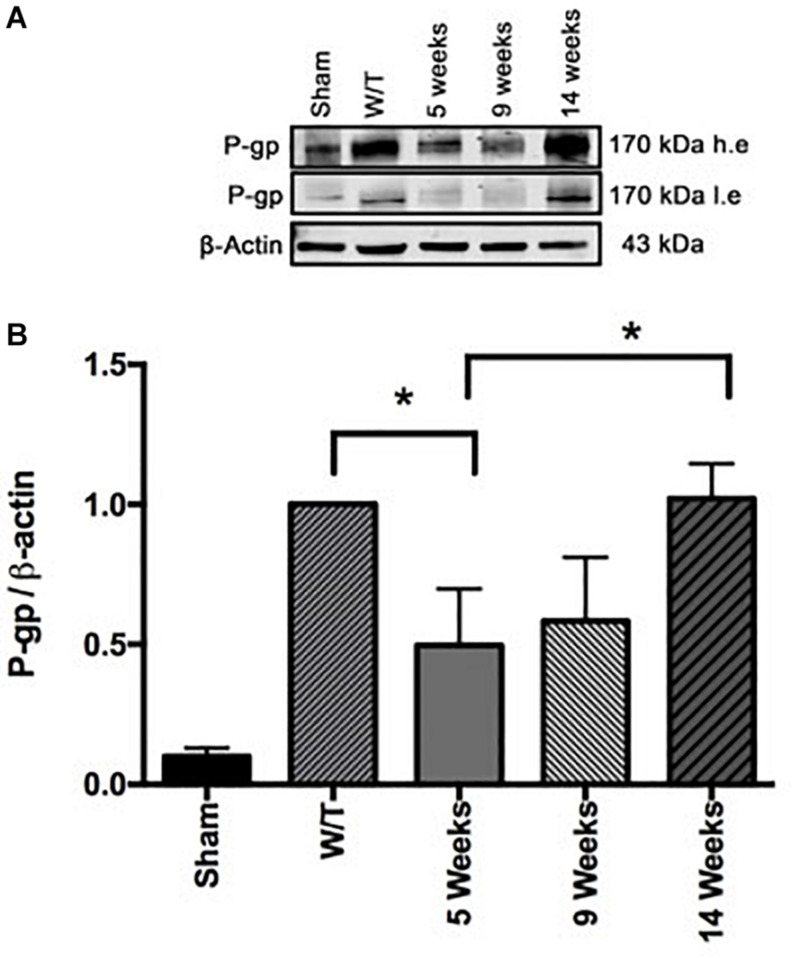
**(A)** Comparison of the levels of P-gp (determined by Western Blot) in the sham-operated rats and two groups of implant-operated animals: one with no drug treatment (W/T) and the other given mifepristone/temozolomide (Mif/Tz) at 5 weeks post-surgery (corresponding to the end of the 3-week drug treatment), and 9, 14 weeks (corresponding to 4 and 9 weeks after the end of drug treatment), h.e., high exposure; l.e., low exposure. **(B)** The densitometer analysis (*n* = 3). Data represent the mean ± SD of three independent experiments. *Significant difference (*p* < 0.05) between the Mif/Tz rats at 5 and 14 weeks.

Evaluation of the expression of P-gp by Western blot at the end of the 3-week drug treatment period (at 5 weeks post-surgery) showed a significantly lower level for Mif/Tz-treated versus untreated rats (Figure 9). This reduced level in the Mif/Tz group was reversed after drug treatment ended, gradually rising until reaching the level of the untreated group at 14 weeks.

### Effect of Mifepristone/Temozolomide on the Level of MGMT During Tumor Recurrence

**FIGURE 10:**
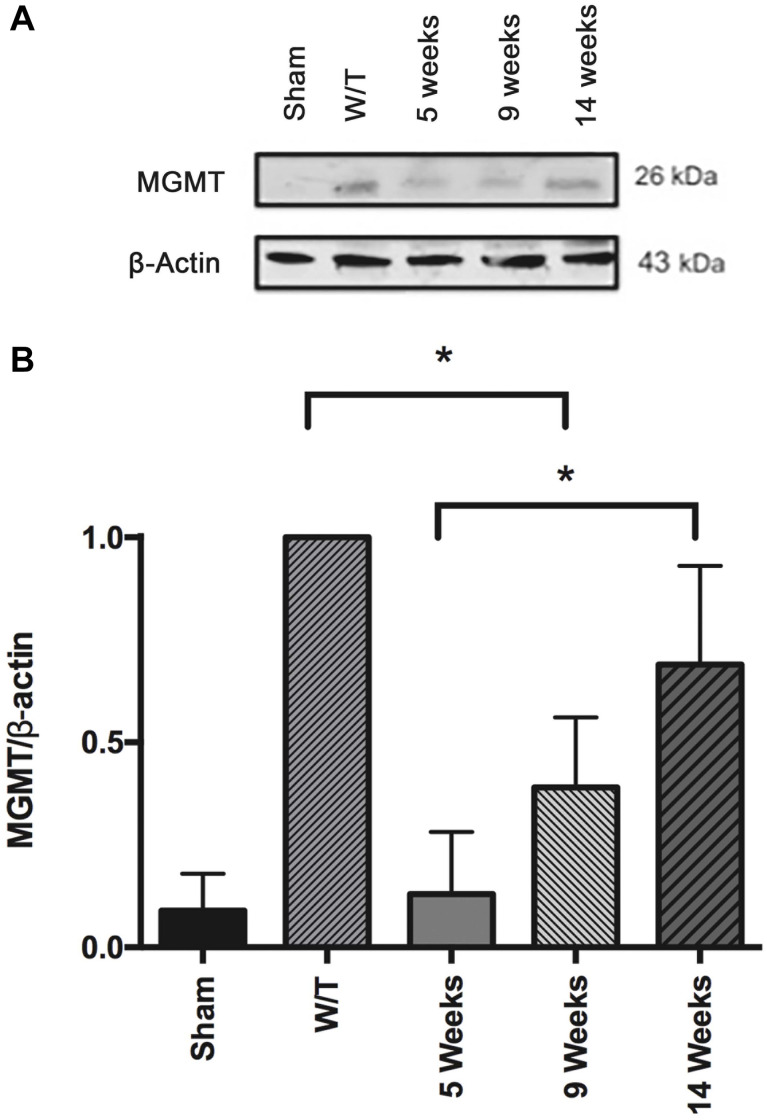
**(A)** Comparison of the level of the DNA repair enzyme, MGMT, determined by Western blot in the sham-operated rats and two groups of implant-operated animals: one with no drug treatment (W/T) and the other given mifepristone/temozolomide (Mif/Tz) at 5 weeks post-surgery (corresponding to the end of the 3-week drug treatment) and 9 and 14 weeks after surgery. A lower level of MGMT was found in the Mif/Tz versus W/T group at 5 weeks post-surgery, an effect that was gradually reversed. **(B)** Densitometer analysis (*n* = 3). Data is expressed as the mean ± SD of three independent experiment. * Significant difference (*p* < 0.05).

At 5 weeks post-surgery, the expression of MGMT was lower in healthy sham rats compared to the untreated animals with implanted cancer cells. This point in time corresponds to the end of the drug treatments, at which time the combination regimen of mifepristone/temozolomide produced a significant decrease in the level of MGMT, in agreement with a our previous report (5). This effect was reversed at weeks 9 and 14, corresponding to the time of tumor recurrence (Figure 10).

## Discussion

Although there have been advances in the treatments of some cancers, the molecules recently developed for glioblastoma therapy have shown little success in improving patient prognosis and survival. Glioblastoma is currently treated with surgery followed by chemotherapy with temozolomide and radiotherapy, resulting in a post-diagnostic median survival time of only 1-2 years. Among the main problems in glioblastoma treatment are rapid proliferation, the limited capacity of drugs to cross the BBB, and other mechanisms related to the resistance of cancer cells to chemotherapy. Thus, new strategies are necessary (1).

It has reported that the antitumor activity of temozolomide is schedule-dependent, with multiple administrations being more effective than a single treatment. In clinical use, the recommended dose of temozolomide is 75 mg/m^2^, daily until with a maximum of 49 doses and in the dose of maintenance of 200 mg/m^2^ given for five consecutive days every 28-day cycle (5/28 days) (9, 10).

The scheme of drug treatments used presently is similar to that used in patients. In our study, temozolomide was administered for only three weeks because it is the average survival time of the rats with the individual treatments.

The dose of temozolomide was calculated based on several reports in the literature and in our previous work. The doses of temozolomide used in the present work is compared to metronomic doses of 2 mg/kg every day for 16 days reported by Kim et al. (25), the authors observed a significant effect on the tumor volume and microvessel density. Moreover there were no signs of toxicity with drug administration, such as body weight loss. Other study also showed similar results using temozolomide at dose of 5 mg/kg/day (26), showing a significant decrease on tumor growth. These results correlate with our previous findings where we used temozolomide 5 mg/kg/day × 21 days, there was a significant decrease on tumor growth measured as the proliferative activity in tumors (5).

In the case of mifepristone, we used a total dose of 150 mg/kg (10mg/kg × 5 days/3 weeks) in rats according with our previous report (5), On the other hand, several reports support that using low dose of the drugs it is more probably to find a synergistic effect when the drugs are combined. This is important in cancer because many studies looking for a synergistic effect more than an additive effect due to the side effects of chemotherapy.

The antihormonal agent mifepristone has been investigated in regard to different types of cancer, both hormone- and non-hormone-dependent (27). Mifepristone acts as an antagonist of progestins, glucocorticoids and androgens through the respective receptors. It reportedly inhibits cell growth in non-hormone-dependent cancer cells, such as MDA-MB-321 (breast cancer) (28) and LNCaP (prostate cancer) (29), which are negative for progesterone, estrogen and androgen receptors.

**FIGURE 11:**
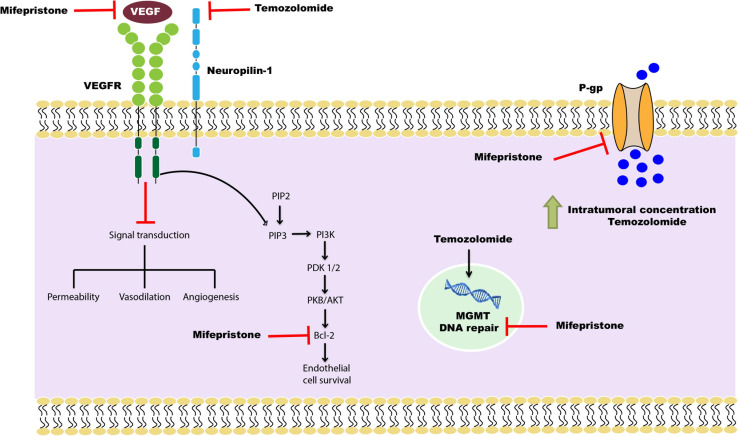
Schematic portrayal of the possible mechanisms of the combination mifepristone/temozolomide treatment that improved the effect found with temozolomide alone. The mechanisms studied were: (1) the inhibition of angiogenesis, measured as reduced levels of VEGF; (2) the attenuation of DNA repair, evaluated as a decrease in MGMT; and (3) the increased capacity of temozolomide to pass through the BBB, assessed as a lower P-gp level and a higher concentration of temozolomide in brain cells. As described in a previous report by our group (5), mifepristone diminishes the level of anti-apoptotic protein Bcl-2 and impedes endothelial cell survival in tumors. This may be the mechanism by which mifepristone/temozolomide herein lowered the level of VEGF. The treatment with mifepristone or temozolomide alone decreased the levels of VEGF to a lesser extent, perhaps by the blockade of autocrine VEGF signaling through specific down-regulation of NRP-1. Additionally, a decline in the expression P-gp was found when administering mifepristone/temozolomide. Thus, this combination treatment may allow for an enhanced intratumoral concentration of temozolomide and contribute to greater tumor cell death. The latter was evidenced by lower tumor proliferation during the drug treatment period. As can be appreciated, mifepristone appears to sensitize glioblastoma cells to the effects of temozolomide.

Previous studies in our laboratory demonstrated the chemo-sensitizing effect of mifepristone in combination with temozolomide in a xenograft and an orthotopic glioma model (5, 30). The current study evaluated two possible molecular mechanisms in this chemo-sensitizing effect: the inhibition of VEGF and CD31 marker to reduce angiogenesis and of P-gp to facilitate the capacity of temozolomide to cross the BBB (Figure 11).

A significant difference in weight was observed between the animals administered mifepristone/temozolomide and those given temozolomide only, mifepristone only, or without treatment animals. This result could be due to the decrease in tumor growth as was observed in the previous reports (5). Typical features of glioblastoma were seen in the H&E images shown; in the group without treatment, there was an increase in hypercellularity and vascular proliferation, which was diminished with the Mif/Tz treatment. A mechanism that has been little explored in cancer-induced weight loss is the modification of metabolic changes involved in cachexia. Cachexia is a complex metabolic disorder that impacts about 80% of patients with advanced cancers (31). Griffith et al. (32) reported body weight loss in glioma patients (32), studies on cachexia symptoms induced by glioblastoma have rarely been reported; Recently Cui et al. (33) demonstrated cachexia manifestations in an orthotopic glioma murine model (33); however, is necessary a metabolic pathway analysis during glioma cachexia. It has been reported that mifepristone impact in cancer cachexia by blocking the interaction of cortisol and induction of zinc-alpha2-glycoprotein (ZAG) expression in adipose tissue (34). On the other hand, cachexia is characterized by systemic inflammation and it has been reported that mifepristone reduced the expression of nuclear transcription factors, including NF-kB (35), a central mediator of pro-inflammatory gene induction. With these antecedents, it is interesting to investigate, in the future, the possible modulation of cachexia by mifepristone/temozolomide treatment.

The tumor microenvironment is known to play a key role in resistance to treatment. In particular, a hypoxic microenvironment is closely related to chemo- and radio-resistance by modulating different mechanisms including angiogenesis (36). Glioma tumors are known to elevate levels of VEGF and its corresponding receptor, the activation of which is related to angiogenesis. Without angiogenesis, tumor growth would be severely limited.

Due to the importance of VEGF in the physiopathology of glioblastoma, one of the strategies to improve patient survival is to diminish its expression. Unfortunately, this strategy has not yet been fruitful. In the current effort, we observed that there was an additive effect by temozolamide and mifepristone in the inhibition of VEGF levels, the Mif/Tz rats exhibited a lower expression of VEGF compared to the other animals with implanted cancer cells, including the untreated, Tz and Mif groups. This results correlated with immunohistochemical studies with CD31 marker, vessel density was decreased in Tz and Mif groups; however, a lower vessel density was observed in Mif/Tz group. Hence, the combined treatment may contribute to an effective strategy for overcoming the resistance of glioblastoma tumors. It is known that the endothelial cells in the vascular bed of tumor are more susceptible to chemotherapeutic agents than resting endothelium, because they have significantly higher proliferation rates than the normal endothelium in the rest of the body. In addition metronomic chemotherapy, which is the continuous administration of the chemotherapeutic agent at a low dose, it exposes endothelial cells in tumor beds to drugs, inducing angiogenesis and apoptosis in endothelial cells before tumor cells (25). Therefore, it is possible that an additive apoptotic effect of Mif/Tz on vascular endothelial cells contribute to antitumor efficacy of the combined drugs.

On the other hand, recently it has been described that temozolomide is able to decrease the expression of VEGF levels at therapeutic or higher doses on U87 glioblastoma cells (37). The authors demonstrated that temozolomide added at doses below its therapeutic dose is not able to induce apoptosis in cells. But it is capable of inducing apoptosis when was introduced in therapeutic dose or above. In our work, the consecutive doses of Mif/Tz administered to the animals could lead to a cumulative dose reaching therapeutic doses that may contribute to an additive effect in the reduction of VEGF levels.

Hernandez-Hernandez et al. described a progesterone-induced increase in the expression of VEGF in the astrocytoma U373 cell line, and a mifepristone-induced reversal of the increase by recruitment of the steroid receptor coactivator (SRC-1) (38). Another possible mechanism leading to a lower level of VEGF is through the regulation of Bcl-2, a protein family composed of cell death regulators. It has been implicated in the differentiation of several cell types, including neuronal, epithelial and hematopoietic cells, as well as in the survival of endothelial cells (39). Karl et al. described pro-angiogenic activity by Bcl-2 based on its ability to activate the NF-κB signaling pathway and elicit expression of the pro-angiogenic CXCL8 and CXCL1 chemokines in endothelial cells (40). According to a previous report by our group, mifepristone reduces Bcl-2 expression in glioma cells (5). Therefore, the diminished VEGF level observed herein could possibly be related to a decrease in Bcl-2 induced by mifepristone.

The BBB, on the other hand, has been the greatest problem for many promising drugs developed to treat glioblastoma. The brain microvascular endothelium is peculiar, characterized by a lack of fenestrations and adherens junctions and by the presence of drug efflux transporters, such as P-glycoprotein (P-gp, Abcb1), the multidrug resistance proteins (MRPs, Abcc1) and breast cancer resistance protein (BCRP, Abcg2) (41). Several researches have focused on the role of inhibition of drug efflux transporters to improve chemotherapy response. P-glycoprotein is the best-characterized molecule of the class of efflux pump transporters, forming part of the BBB by removing drugs from the brain. This protein is expressed by endothelial cells in both healthy brain tissue and gliomas, and a key role has been attribute to it in the chemoresistance of several types of tumors (e.g., gliomas) (42). Consequently, it probably contributes to a low concentration of temozolomide in glioma tumor cells.

The present study found a significant drop in the level of P-gp in the Mif/Tz group. A decrease the levels of P-gp in patients should be able to enhance the intracellular distribution of temozolomide in brain tissue and trigger greater tumor cell death. Various transcription factors (in addition to transcriptional/translational regulation) are involved in regulation of efflux pump transporters (43). This protein is known to be regulated by a nuclear receptor, the pregnane X receptor (PXR) (44–46), which mediates the activation of several genes by xenobiotics, including several ABC transporters. Although the *PXR* promoter has not yet been characterized, dexamethasone is reported to boost *PXR* mRNA levels in primary cultures of human hepatocytes and rat hepatoma H4IIE cells, an effect blocked by mifepristone, suggesting that the GR pathway is involved in the regulation of these transporters (47, 48).

On an other hand it has been reported that glioblastoma is characterized by aberrant activation of inflammatory responses; von Wedel-Parlow et al., reported that the pro-inflammatory cytokines interleukin-1 (IL-1b) and tumor necrosis factor-a (TNF-alpha) affect the expression of cerebral ABC-transporters in primary endothelial cells, the anti-inflammatory glucocorticoid hydrocortisone leads to a induction of Abcg2 (BCRP) and Abcc1 (MRP) mRNA in microvascular endothelial cells whereas Abcb1 (P-gp)gene expression is down-regulated (49). It has been reported that mifepristone decreased the levels of of TNF-alpha in rats exposed to Paraquat (50), and in endometrial epithelial and stromal cells reduced the secretion of IL-6 and TNF-alpha (51). However, more research is necessary to better understand the regulation and the role of mifepristone in efflux pump transporters.

Other strategy to improve treatment response is blocking the drug efflux transporters. Gooijer et al., reported an accumulation about 1.5 fold more of temozolomide in the brain by P-gp and BCRP inhibitors (52). These drug efflux transporters might be possible target of mifepristone to improve the efficacy of temozolomide against glioblastoma.

In the current contribution, the participation of mifepristone in the inhibition of drug efflux transporters was explored indirectly by evaluating the intracerebral concentration of temozolomide, representing a direct and indirect approach, respectively. The Mif/Tz rats exhibited a significantly lower level of P-gp and an increased intracerebral concentration of temozolomide compared to the Tz group. These results are consistent with the findings published by various authors. Mifepristone inhibits the activity of P-gp in a gastric cell line SGC7901/VCR (37) and in KG1a leukemia cells (23), enhances doxorubicin cellular accumulation in resistant human K562 leukemia cells (53), and increases the concentration of cisplatin in the tumors of mice given a combined cisplatin/mifepristone treatment (54). Hence, the blocking of drug efflux transporters by mifepristone could possibly increase the intracellular bioavailability of temozolomide in brain and tumor cells of patients, which should improve the therapeutic response.

Other drug efflux transporters that plays an important role in treatment resistance is MRP and blocking it could be an important strategy, it has been reported that mifepristone exhibited selective MRP1 inhibition (55). Hence, the blocking of drug efflux transporters by mifepristone could possibly increase the concentration of temozolomide in brain and consequently tumor cells can increase the disposition to drug.

In the second part of the present investigation, tumor growth after of the mifepristone/temozolomide treatment was monitored with a microPET/CT scanner measuring 18F-FLT uptake (Figure 6). There was a remarkable decrease at 7 week post-implantation with molecular imaging showing no proliferative activity. Afterward, new proliferation was observed at 9 week post-surgery, indicating tumor relapse. Nevertheless, the animals maintained a constant body weight and the proliferative activity did not rise by the next measurement at 14 weeks. The H&E images shown in the group at 7 weeks, there was a decrease in hypercellularity and vascular proliferation. However, after the end of the drug treatment, an infiltration of neoplastic cells with a hyperchromatic nucleus was observed again, in addition to an increase in the mitotic index and pseudopalisading. Despite being observed again these typical features of glioblastoma, which are associated with a poor prognosis, the animals survided longer. These results were corroborated with molecular images where it was observed tumor recurrence at week 9. Moreover, 70% of rats given mifepristone/temozolomide survived 60–70 days and approximately 30% survived over 100 days. In glioblastoma patients, a relapsed tumor inevitably causes 100% mortality.

Another molecular mechanism explored presently was the effect of the Mif/Tz treatment on MGMT, which is related to DNA repair in tumor cells. Glioblastoma stem cells are reported to express high levels of MGMT (56) and P-gp, in both cases generating more resistance to temozolomide, and therefore a greater probability of tumor relapse (57). Several studies have suggested that stem cells may be responsible for resistance and recurrence in glioblastoma. In such a case, a challenge in the treatment of glioblastoma would be the removal not only of the tumor cells, but also the glioblastoma stem cells.

O6-methylguanine-DNA-methyltransferase was found to significantly decrease by the end of the 3-week Mif/Tz treatment, thus confirming a previous finding by our group. Indeed, MGMT followed the same pattern as VEGF and P-gp. All three parameters were found to decrease during the Mif/Tz treatment, and then increase afterward. Within 14 weeks, all three of these molecules reached levels similar to the control group. In our study drug treatment were given only by 3 weeks; we did not observed adverse effects associated with the administration of mifepristone. The decrease of weight gain in the animals was due to implantation of tumor cells. In according to the literature, several clinical studies of mifepristone in patients with breast cancer (58), meningioma (59), and non-small cell lung cancer (60) have demonstrated that mifepristone has tolerable side effect, including nausea, lethargy, anorexia, fatigue, and hot flashes; even when mifepristone has been taken daily for long periods of time, it has mild adverse effects; therefore, the long-term administration of mifepristone may be feasible and well tolerated; we proposed in the near future to test this possibility and to evaluate whether mifepristone offers greater benefits during tumor recurrence. According to the current results, mifepristone could possibly contribute to the modulation of tumor relapse in glioblastoma by decreasing the levels of VEGF, MGMT, and P-gp. Further research is needed to explore other mechanisms of drug resistance of glioblastoma tumors.

## Conclusion

Mifepristone herein improved the effect of temozolomide. The mifepristone/temozolomide combination produced a sharply lower expression of VEGF, CD31, P-gp, and MGMT compared to the other groups with implanted cancer cells, including the untreated animals and those given mifepristone or temozolomide alone. Moreover, the combination treatment increased the intracerebral concentration of temozolomide and diminished tumor proliferation. The present results strongly suggest that mifepristone could serve as part of a strategy to overcome the resistance of glioblastoma tumors to temozolomide. Future research is required to determine whether the mifepristone/temozolomide regimen can regulate glioma stem cells and inhibit the mechanisms related to tumor relapse.

## Data Availability Statement

The datasets generated for this study are available on request to the corresponding author.

## Ethics Statement

The animal study was reviewed and approved by Ethics Committee of the “Instituto Nacional de Cancerología” (INCan, Mexico City, Mexico) (Ref No. 010-17-IBICB601-10).

## Author Contributions

ML-M participated in the experimental procedures for tumor cell implantation, helped with data processing, and performed the analysis of the results. SL-Z and MR-G designed the histological experiments. IV-L contributed to the LC/MS experiments for quantification of temozolomide in brain tissue. LM carried out the evaluation of the tumor growth by molecular imaging. PG-L planned and supervised the entire study. All authors read and approved the final version of the manuscript.

## Conflict of Interest

The authors declare that the research was conducted in the absence of any commercial or financial relationships that could be construed as a potential conflict of interest.

## References

[1] Johnson DR O’Neill BP. Glioblastoma survival in the United States before and during the temozolomide era. *J Neurooncol.* (2012) 107:359–64. 10.1007/s11060-011-0749-4 22045118

[2] Thakkar JP Dolecek TA Horbinski C Ostrom QT Lightner DD Barnholtz-Sloan JS Epidemiologic and molecular prognostic review of glioblastoma. *Cancer Epidemiol Biomarkers Prev.* (2014) 23:1985–96. 10.1158/1055-9965.EPI-14-0275 25053711 PMC4185005

[3] Witthayanuwat S Pesee M Supaadirek C Supakalin N Thamronganantasakul K Krusun S. Survival analysis of glioblastoma multiforme. *Asian Pac J Cancer Prev.* (2018) 19:2613–7. 10.22034/APJCP.2018.19.9.2613 30256068 PMC6249474

[4] Wang H Xu T Jiang Y Xu H Yan Y Fu D The challenges and the promise of molecular targeted therapy in malignant gliomas. *Neoplasia.* (2015) 17:239–55. 10.1016/j.neo.2015.02.002 25810009 PMC4372648

[5] Llaguno-Munive M Romero-Pina M Serrano-Bello J Medina LA Uribe-Uribe N Salazar AM Mifepristone overcomes tumor resistance to temozolomide associated with DNA damage repair and apoptosis in an orthotopic model of glioblastoma. *Cancers.* (2019) 11:16. 10.3390/cancers11010016 30583528 PMC6356343

[6] Nishida N Yano H Nishida T Kamura T Kojiro M. Angiogenesis in cancer. *Vasc Health Risk Manag.* (2006) 2:213–9. 10.2147/vhrm.2006.2.3.213 17326328 PMC1993983

[7] Machein MR Plate KH. VEGF in brain tumors. *J Neurooncol.* (2000) 50:109–20. 11245271 10.1023/a:1006416003964

[8] Kaur B Khwaja FW Severson EA Matheny SL Brat DJ Van Meir EG. Hypoxia and the hypoxia-inducible-factor pathway in glioma growth and angiogenesis. *Neuro Oncol.* (2005) 7:134–53. 10.1215/S1152851704001115 15831232 PMC1871894

[9] Gilbert MR Dignam JJ Armstrong TS Wefel JS Blumenthal DT Vogelbaum MA A randomized trial of bevacizumab for newly diagnosed glioblastoma. *N Engl J Med.* (2014) 370:699–708. 10.1056/NEJMoa1308573 24552317 PMC4201043

[10] Chinot OL Wick W Mason W Henriksson R Saran F Nishikawa R Bevacizumab plus radiotherapy-temozolomide for newly diagnosed glioblastoma. *N Engl J Med.* (2014) 370:709–22. 10.1056/NEJMoa1308345 24552318

[11] Louis DN. Molecular pathology of malignant gliomas. *Annu Rev Pathol.* (2006) 1:97–117. 10.1146/annurev.pathol.1.110304.100043 18039109

[12] Schaich M Kestel L Pfirrmann M Robel K Illmer T Kramer M A MDR1 (ABCB1) gene single nucleotide polymorphism predicts outcome of temozolomide treatment in glioblastoma patients. *Ann Oncol.* (2009) 20:175–81. 10.1093/annonc/mdn548 18687982

[13] Agarwal S Manchanda P Vogelbaum MA Ohlfest JR Elmquist WF. Function of the blood-brain barrier and restriction of drug delivery to invasive glioma cells: findings in an orthotopic rat xenograft model of glioma. *Drug Metab Dispos.* (2013) 41:33–9. 10.1124/dmd.112.048322 23014761 PMC3533422

[14] Da Ros M De Gregorio V Iorio AL Giunti L Guidi M de Martino M Glioblastoma chemoresistance: the double play by microenvironment and blood-brain barrier. *Int J Mol Sci.* (2018) 19:2879. 10.3390/ijms19102879 30248992 PMC6213072

[15] Gaddy VT Barrett JT Delk JN Kallab AM Porter AG Schoenlein PV. Mifepristone induces growth arrest, caspase activation, and apoptosis of estrogen receptor-expressing, antiestrogen-resistant breast cancer cells. *Clin Cancer Res.* (2004) 10:5215–25. 10.1158/1078-0432.CCR-03-0637 15297425

[16] Liu R Shi P Nie Z Liang H Zhou Z Chen W Mifepristone suppresses basal triple-negative breast cancer stem cells by down-regulating KLF5 expression. *Theranostics.* (2016) 6:533–44. 10.7150/thno.14315 26941846 PMC4775863

[17] Segovia-Mendoza M Jurado R Mir R Medina LA Prado-Garcia H Garcia-Lopez P. Antihormonal agents as a strategy to improve the effect of chemo-radiation in cervical cancer: in vitro and in vivo study. *BMC Cancer.* (2015) 15:21. 10.1186/s12885-015-1016-4 25622528 PMC4311459

[18] Moe BT Vereide AB Orbo A Jaeger R Sager G. Levonorgestrel, medroxyprogesterone and progesterone cause a concentration-dependent reduction in endometrial cancer (Ishikawa) cell density, and high concentrations of progesterone and mifepristone act in synergy. *Anticancer Res.* (2009) 29:1047–52. 19414344

[19] Goyeneche AA Caron RW Telleria CM. Mifepristone inhibits ovarian cancer cell growth in vitro and in vivo. *Clin Cancer Res.* (2007) 13:3370–9. 10.1158/1078-0432.CCR-07-0164 17545545 PMC2505183

[20] Ritch SJ Brandhagen BN Goyeneche AA Telleria CM. Advanced assessment of migration and invasion of cancer cells in response to mifepristone therapy using double fluorescence cytochemical labeling. *BMC Cancer.* (2019) 19:376. 10.1186/s12885-019-5587-3 31014286 PMC6480622

[21] Touat M Lombardi G Farina P Kalamarides M Sanson M. Successful treatment of multiple intracranial meningiomas with the antiprogesterone receptor agent mifepristone (RU486). *Acta Neurochir.* (2014) 156:1831–5. 10.1007/s00701-014-2188-4 25078073

[22] Check JH Wilson C Cohen R Sarumi M. Evidence that mifepristone, a progesterone receptor antagonist, can cross the blood brain barrier and provide palliative benefits for glioblastoma multiforme grade IV. *Anticancer Res.* (2014) 34:2385–8. 24778047

[23] Fardel O Courtois A Drenou B Lamy T Lecureur V le Prise PY Inhibition of P-glycoprotein activity in human leukemic cells by mifepristone. *Anticancer Drugs.* (1996) 7:671–7. 10.1097/00001813-199608000-00008 8913436

[24] Li DQ Wang ZB Bai J Zhao J Wang Y Hu K Reversal of multidrug resistance in drug-resistant human gastric cancer cell line SGC7901/VCR by antiprogestin drug mifepristone. *World J Gastroenterol.* (2004) 10:1722–5. 10.3748/wjg.v10.i12.1722 15188493 PMC4572256

[25] Kim JT Kim JS Ko KW Kong DS Kang CM Kim MH Metronomic treatment of temozolomide inhibits tumor cell growth through reduction of angiogenesis and augmentation of apoptosis in orthotopic models of gliomas. *Oncol Rep.* (2006) 16:33–9. 16786120

[26] Sun C Yu Y Wang L Wu B Xia L Feng F Additive antiangiogenesis effect of ginsenoside Rg3 with low-dose metronomic temozolomide on rat glioma cells both in vivo and in vitro. *J Exp Clin Cancer Res.* (2016) 35:32. 10.1186/s13046-015-0274-y 26872471 PMC4752767

[27] Tieszen CR Goyeneche AA Brandhagen BN Ortbahn CT Telleria CM. Antiprogestin mifepristone inhibits the growth of cancer cells of reproductive and non-reproductive origin regardless of progesterone receptor expression. *BMC Cancer.* (2011) 11:207. 10.1186/1471-2407-11-207 21619605 PMC3125282

[28] Liang Y Hou M Kallab AM Barrett JT El Etreby F Schoenlein PV. Induction of antiproliferation and apoptosis in estrogen receptor negative MDA-231 human breast cancer cells by mifepristone and 4-hydroxytamoxifen combination therapy: a role for TGFbeta1. *Int J Oncol.* (2003) 23:369–80. 12851686

[29] El Etreby MF Liang Y Lewis RW. Induction of apoptosis by mifepristone and tamoxifen in human LNCaP prostate cancer cells in culture. *Prostate.* (2000) 43:31–42. 10.1002/(sici)1097-0045(20000401)43:1<31::aid-pros5>3.0.co;2-# 10725863

[30] Llaguno-Munive M Medina LA Jurado R Romero-Pina M Garcia-Lopez P. Mifepristone improves chemo-radiation response in glioblastoma xenografts. *Cancer Cell Int.* (2013) 13:29. 10.1186/1475-2867-13-29 23530939 PMC3626552

[31] Pin F Barreto R Couch ME Bonetto A O’Connell TM. Cachexia induced by cancer and chemotherapy yield distinct perturbations to energy metabolism. *J Cachexia Sarcopenia Muscle.* (2019) 10:140–54. 10.1002/jcsm.12360 30680954 PMC6438345

[32] Griffith JL Hochberg FH. Anorexia and weight loss in glioma patients. *Psychosomatics.* (1988) 29:335–7. 10.1016/S0033-3182(88)72373-7 2841711

[33] Cui P Shao W Huang C Wu CJ Jiang B Lin D. Metabolic derangements of skeletal muscle from a murine model of glioma cachexia. *Skelet Muscle.* (2019) 9:3. 10.1186/s13395-018-0188-4 30635036 PMC6330447

[34] Russell ST Tisdale MJ. The role of glucocorticoids in the induction of zinc-alpha2-glycoprotein expression in adipose tissue in cancer cachexia. *Br J Cancer.* (2005) 92:876–81. 10.1038/sj.bjc.6602404 15714206 PMC2361908

[35] Boopalan T Arumugam A Parada J Saltzstein E Lakshmanaswamy R. Receptor activator for nuclear factor-kappaB ligand signaling promotes progesterone-mediated estrogen-induced mammary carcinogenesis. *Cancer Sci.* (2015) 106:25–33. 10.1111/cas.12571 25412610 PMC4317778

[36] Tate MC Aghi MK. Biology of angiogenesis and invasion in glioma. *Neurotherapeutics.* (2009) 6:447–57. 10.1016/j.nurt.2009.04.001 19560735 PMC5084181

[37] Mirabdaly S Elieh Ali Komi D Shakiba Y Moini A Kiani A. Effects of temozolomide on U87MG glioblastoma cell expression of CXCR4, MMP2, MMP9, VEGF, anti-proliferatory cytotoxic and apoptotic properties. *Mol Biol Rep.* (2020) 47:1187–97. 10.1007/s11033-019-05219-2 31897867

[38] Hernandez-Hernandez OT Gonzalez-Garcia TK Camacho-Arroyo I. Progesterone receptor and SRC-1 participate in the regulation of VEGF, EGFR and Cyclin D1 expression in human astrocytoma cell lines. *J Steroid Biochem Mol Biol.* (2012) 132:127–34. 10.1016/j.jsbmb.2012.04.005 22542550

[39] Mabeta P. Oncosuppressors and oncogenes: role in haemangioma genesis and potential for therapeutic targeting. *Int J Mol Sci.* (2018) 19:1192. 10.3390/ijms19041192 29652858 PMC5979526

[40] Karl E Warner K Zeitlin B Kaneko T Wurtzel L Jin T Bcl-2 acts in a proangiogenic signaling pathway through nuclear factor-kappaB and CXC chemokines. *Cancer Res.* (2005) 65:5063–9. 10.1158/0008-5472.CAN-05-0140 15958549

[41] Ueno M Nakagawa T Wu B Onodera M Huang CL Kusaka T Transporters in the brain endothelial barrier. *Curr Med Chem.* (2010) 17:1125–38. 10.2174/092986710790827816 20175745

[42] Toth K Vaughan MM Peress NS Slocum HK Rustum YM. MDR1 P-glycoprotein is expressed by endothelial cells of newly formed capillaries in human gliomas but is not expressed in the neovasculature of other primary tumors. *Am J Pathol.* (1996) 149:853–8. 8780389 PMC1865160

[43] Scotto KW. Transcriptional regulation of ABC drug transporters. *Oncogene.* (2003) 22:7496–511. 10.1038/sj.onc.1206950 14576854

[44] Whyte-Allman SK Hoque MT Jenabian MA Routy JP Bendayan R. Xenobiotic nuclear receptors pregnane X receptor and constitutive androstane receptor regulate antiretroviral drug efflux transporters at the blood-testis barrier. *J Pharmacol Exp Ther.* (2017) 363:324–35. 10.1124/jpet.117.243584 28970358

[45] Bauer B Hartz AM Fricker G Miller DS. Pregnane X receptor up-regulation of P-glycoprotein expression and transport function at the blood-brain barrier. *Mol Pharmacol.* (2004) 66:413–9. 10.1124/mol.66.3 15322232

[46] Narang VS Fraga C Kumar N Shen J Throm S Stewart CF Dexamethasone increases expression and activity of multidrug resistance transporters at the rat blood-brain barrier. *Am J Physiol Cell Physiol.* (2008) 295:C440–50. 10.1152/ajpcell.00491.2007 18524938 PMC2518425

[47] Kliewer SA Goodwin B Willson TM. The nuclear pregnane X receptor: a key regulator of xenobiotic metabolism. *Endocr Rev.* (2002) 23:687–702. 10.1210/er.2001-0038 12372848

[48] Pascussi JM Drocourt L Fabre JM Maurel P Vilarem MJ. Dexamethasone induces pregnane X receptor and retinoid X receptor-alpha expression in human hepatocytes: synergistic increase of CYP3A4 induction by pregnane X receptor activators. *Mol Pharmacol.* (2000) 58:361–72. 10.1124/mol.58.2.361 10908304

[49] von Wedel-Parlow M Wolte P Galla HJ. Regulation of major efflux transporters under inflammatory conditions at the blood-brain barrier in vitro. *J Neurochem.* (2009) 111:111–8. 10.1111/j.1471-4159.2009.06305.x 19656257

[50] Hong GL Cai QQ Tan JP Jiang XZ Zhao GJ Wu B Mifepristone-inducible recombinant adenovirus attenuates paraquat-induced lung injury in rats. *Hum Exp Toxicol.* (2015) 34:32–43. 10.1177/0960327114532381 24812154

[51] Che X Wang J He J Guo X Li T Zhang X. The new application of mifepristone in the relief of adenomyosis-caused dysmenorrhea. *Int J Med Sci.* (2020) 17:224–33. 10.7150/ijms.39252 32038106 PMC6990887

[52] de Gooijer MC de Vries NA Buckle T Buil LCM Beijnen JH Boogerd W Improved brain penetration and antitumor efficacy of temozolomide by inhibition of ABCB1 and ABCG2. *Neoplasia.* (2018) 20:710–20. 10.1016/j.neo.2018.05.001 29852323 PMC6030392

[53] Lecureur V Fardel O Guillouzo A. The antiprogestatin drug RU 486 potentiates doxorubicin cytotoxicity in multidrug resistant cells through inhibition of P-glycoprotein function. *Lett.* (1994) 335:187–91. 10.1016/0014-5793(94)01186-9 7982498

[54] Jurado R Lopez-Flores A Alvarez A Garcia-Lopez P. Cisplatin cytotoxicity is increased by mifepristone in cervical carcinoma: an in vitro and in vivo study. *Oncol Rep.* (2009) 22:1237–45. 10.3892/or_00000560 19787245

[55] Sampson A Peterson BG Tan KW Iram SH. Doxorubicin as a fluorescent reporter identifies novel MRP1 (ABCC1) inhibitors missed by calcein-based high content screening of anticancer agents. *Biomed Pharmacother.* (2019) 118:109289. 10.1016/j.biopha.2019.109289 31401398

[56] Pistollato F Abbadi S Rampazzo E Persano L Della Puppa A Frasson C Intratumoral hypoxic gradient drives stem cells distribution and MGMT expression in glioblastoma. *Stem Cells.* (2010) 28:851–62. 10.1002/stem.415 20309962

[57] Eramo A Ricci-Vitiani L Zeuner A Pallini R Lotti F Sette G Chemotherapy resistance of glioblastoma stem cells. *Cell Death Differ.* (2006) 13:1238–41. 10.1038/sj.cdd.4401872 16456578

[58] Klijn JG de Jong FH Bakker GH Lamberts SW Rodenburg CJ Alexieva-Figusch J. Antiprogestins, a new form of endocrine therapy for human breast cancer. *Cancer Res.* (1989) 49:2851–6. 2720645

[59] Grunberg SM Weiss MH Russell CA Spitz IM Ahmadi J Sadun A Long-term administration of mifepristone (RU486): clinical tolerance during extended treatment of meningioma. *Cancer Invest.* (2006) 24:727–33. 10.1080/07357900601062339 17162554

[60] Check JH Check D Poretta T. Mifepristone extends both length and quality of life in a patient with advanced non-small cell lung cancer that has progressed despite chemotherapy and a check-point inhibitor. *Anticancer Res.* (2019) 39:1923–6. 10.21873/anticanres.13301 30952734

