# Corrigendum: Mifepristone as a Potential Therapy to Reduce Angiogenesis and P-Glycoprotein Associated With Glioblastoma Resistance to Temozolomide

**DOI:** 10.3389/fonc.2021.675806

**Published:** 2021-03-22

**Authors:** Monserrat Llaguno-Munive, Sebastián León-Zetina, Inés Vazquez-Lopez, María del Pilar Ramos-Godinez, Luis A. Medina, Patricia Garcia-Lopez

**Affiliations:** ^1^ Laboratorio de Farmacología, Subdirección de Investigación Básica, Instituto Nacional de Cancerología, Mexico City, Mexico; ^2^ Posgrado en Ciencias Biomédicas, Universidad Nacional Autónoma de México, Mexico City, Mexico; ^3^ Departamento de Patología Quirúrgica, Instituto Nacional de Cancerología, Mexico City, Mexico; ^4^ Unidad de Investigación Biomédica en Cáncer INCan-UNAM, Instituto Nacional de Cancerología, Mexico City, Mexico; ^5^ Instituto de Física, Universidad Nacional Autónoma de México, Mexico City, Mexico

**Keywords:** glioblastoma, temozolomide, mifepristone, drug resistance, angiogenesis, P-gp

In the original article, there was a mistake in **Figure 2**. Wrong microscopy photographs were included in the Sham and Mif/Tz 3-week groups. The corrected **Figure 2** appears below.

**Figure 2:**
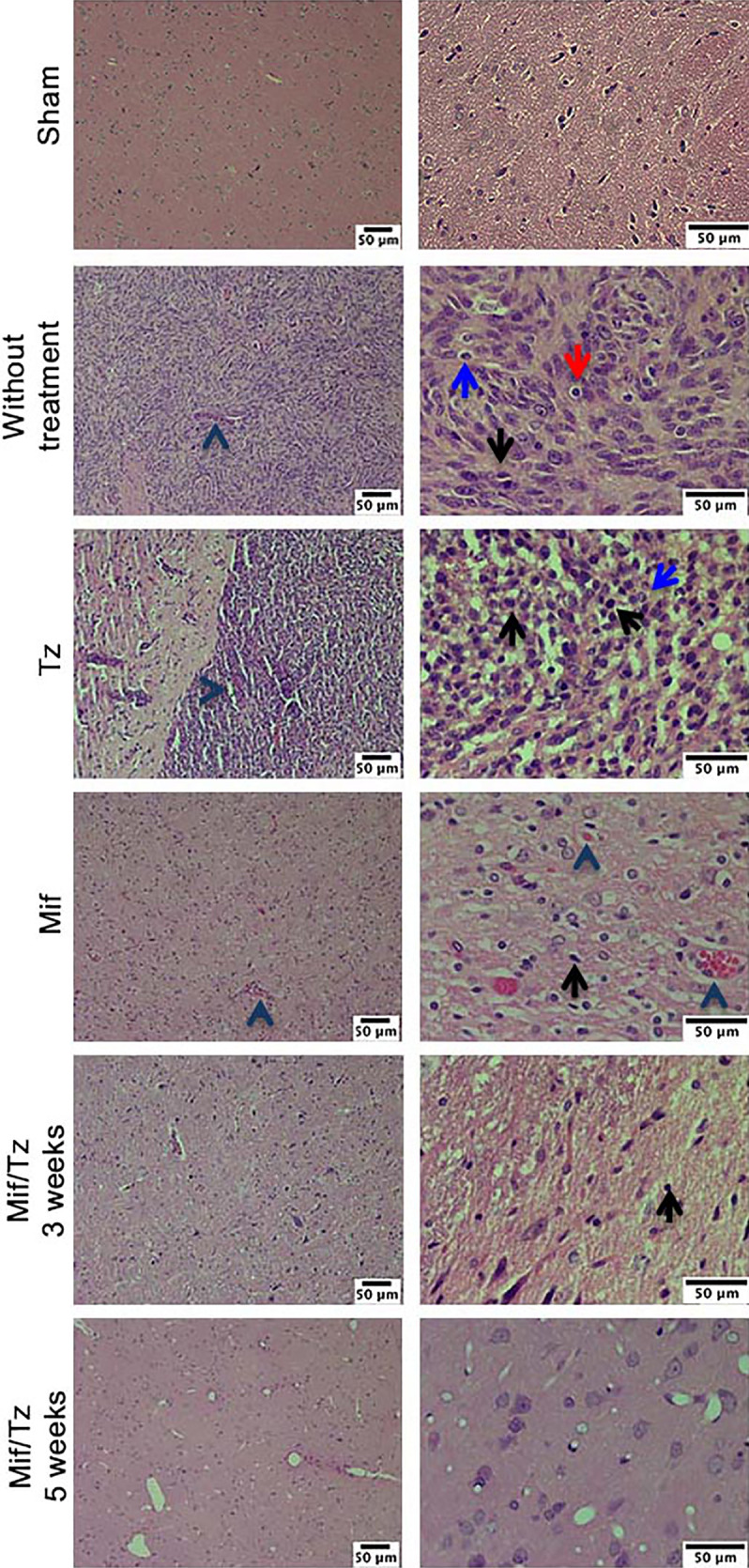
Hematoxylin and eosin (H&E) staining analysis of glioma tissue. Hyperbasophilic cells (black arrow), hyperchromatics cells (red arrow), vessel proliferation (arrowhead), mitosis (blue arrow). The images are representative of three animals per treatment Scale bars = 50 mm.

The authors apologize for this mistake and state that this does not change the article’s scientific discussion and conclusions in any way. The original article has been updated.

